# Absent otoacoustic emissions predict otitis media in young Aboriginal children: A birth cohort study in Aboriginal and non-Aboriginal children in an arid zone of Western Australia

**DOI:** 10.1186/1471-2431-8-32

**Published:** 2008-08-28

**Authors:** Deborah Lehmann, Sharon Weeks, Peter Jacoby, Dimity Elsbury, Janine Finucane, Annette Stokes, Ruth Monck, Harvey Coates, the Kalgoorlie Otitis Media Research Project Team

**Affiliations:** 1 Telethon Institute for Child Health Research, Centre for Child Health Research, the University of Western Australia, Australia; 2 Professional Hearing Services, South Perth, Western Australia, Australia; 3 School of Paediatrics and Child Health, the University of Western Australia, Australia

## Abstract

**Background:**

Otitis media (OM) is the most common paediatric illness for which antibiotics are prescribed. In Australian Aboriginal children OM is frequently asymptomatic and starts at a younger age, is more common and more likely to result in hearing loss than in non-Aboriginal children. Absent transient evoked otoacoustic emissions (TEOAEs) may predict subsequent risk of OM.

**Methods:**

100 Aboriginal and 180 non-Aboriginal children in a semi-arid zone of Western Australia were followed regularly from birth to age 2 years. Tympanometry was conducted at routine field follow-up from age 3 months. Routine clinical examination by an ENT specialist was to be done 3 times and hearing assessment by an audiologist twice. TEOAEs were measured at ages <1 and 1–2 months. Cox proportional hazards model was used to investigate the association between absent TEOAEs and subsequent risk of OM.

**Results:**

At routine ENT specialist clinics, OM was detected in 55% of 184 examinations in Aboriginal children and 26% of 392 examinations in non-Aboriginal children; peak prevalence was 72% at age 5–9 months in Aboriginal children and 40% at 10–14 months in non-Aboriginal children. Moderate-severe hearing loss was present in 32% of 47 Aboriginal children and 7% of 120 non-Aboriginal children aged 12 months or more.

TEOAE responses were present in 90% (46/51) of Aboriginal children and 99% (120/121) of non-Aboriginal children aged <1 month and in 62% (21/34) and 93% (108/116), respectively, in Aboriginal and non-Aboriginal children at age 1–2 months. Aboriginal children who failed TEOAE at age 1–2 months were 2.6 times more likely to develop OM subsequently than those who passed.

Overall prevalence of type B tympanograms at field follow-up was 50% (n = 78) in Aboriginal children and 20% (n = 95) in non-Aboriginal children.

**Conclusion:**

The burden of middle ear disease is high in all children, but particularly in Aboriginal children, one-third of whom suffer from moderate-severe hearing loss. In view of the frequently silent nature of OM, every opportunity must be taken to screen for OM. Measurement of TEOAEs at age 1–2 months to identify children at risk of developing OM should be evaluated in a routine health service setting.

## Background

In industrialised countries otitis media (OM) is the most common paediatric illness for which medical advice is sought and antibiotics are prescribed [1]. OM can lead to impaired hearing, which can seriously affect early language development, performance at school, and subsequent employment and social integration in adulthood. Repeated antibiotic treatment for OM contributes to the increasing levels of antibiotic resistance worldwide [2].

In the general population, OM incidence peaks at age 6–18 months and 3–17% of children suffer ≥ 3 attacks of acute OM annually. The prevalence of OM with effusion (OME) is ~12% at age 1 year [3]. In Western Australia (WA), OM is the second most common reason for paediatric hospital admission [4]. There are no population-based data on the burden of OM in the general population of Australia other than a nationwide study conducted 30 years ago [5].

Aboriginal Australians are the most disadvantaged sector of Australian society [6]. The enormous burden of OM in Australian Aboriginal children contributes to lifelong social disadvantage. Compared with non-Aboriginal children, OM in Aboriginal children is more common, starts at a younger age, is more likely to result in hearing loss and is associated with early onset of upper respiratory tract bacterial carriage [7-11]. The disease may be asymptomatic until purulent ear discharge is visible [8] and so treatment may not be sought until late in the disease process [12]. A recent study in remote communities in the Northern Territory (NT) of Australia showed that 91% of Aboriginal children aged 6–30 months had current clinical signs of OM and that tympanic membrane (TM) perforation rates varied between communities from 0% to 60% [11]. Data on the burden of OM in young Aboriginal children living in urban areas are sparse [5,13]. Despite the enormous burden of disease, there is currently no routine screening for ear health in preschool-age children in Western Australia. Thus, many Aboriginal children reach school age having had recurrent or continuous ear infections with serious consequences, in particular hearing loss and impaired language development. This results in poor educational attainment and behavioural problems, perpetuating the cycle of ill health, poverty and social exclusion faced by many Aboriginal people. In view of the early onset of disease which is frequently asymptomatic in Aboriginal children, we need appropriate methods of identifying children at high risk of OM in early infancy. Clinical diagnosis of OM in young children is difficult. Therefore a simple affordable tool that can be used at the primary health care level is needed to identify those in need of prompt treatment to avoid the serious consequences of tympanic membrane perforation and hearing loss.

Tympanometry is a standard method used to detect middle ear effusion and OM. Impedance audiometry (tympanometry) is normally conducted using a 226 Hz probe and is effective in people over the age of 6 months [14,15]. Before age 6 months, a 1000 Hz probe tone improves sensitivity of the test and a screening instrument with 1000 Hz probe tone has recently become available.

Measurement of otoacoustic emissions (OAEs) offers an alternative to high-frequency tympanometry to assess middle ear function in early infancy [16]. OAEs originating in the cochlea are low-level sounds in response to a given stimulus and can be measured in the outer ear. Transient evoked OAEs (TEOAEs) are used widely to identify sensorineural hearing loss in neonates, but OAEs may be absent as a result of fluid in the middle ear of young infants [17-19]. Furthermore, absent OAEs in young children may identify children at risk of OM in the future [20]. However, a recent cohort study of American Indian children followed from birth to age 2 years found no association between absent OAEs in the first month of life and subsequent risk of OM [21]. There are no other data on OAEs for indigenous populations and to our knowledge no studies have investigated the association between presence or absence of TEOAEs in early infancy after the neonatal period (without the use of other assessments of middle ear disease such as otoscopy) and subsequent risk of OM. Measurement of OAEs in the postneonatal period may offer a simple tool for use by primary health care workers to identify a high-risk group of children.

Between 1999 and 2005, we undertook a study in the Kalgoorlie-Boulder area of WA, a semi-arid region approximately 600 km east of the state capital, Perth, to investigate the causal pathways to OM in Aboriginal and non-Aboriginal children. The study aimed to identify the most important, avoidable risk factors in order to develop appropriate interventions [22]. As expected, we found that, compared to non-Aboriginal participants, Aboriginal mothers were younger, smoked more and had poorer educational outcomes, mother and fathers were less likely to be employed and families lived in more crowded conditions [22]. In this paper we report for the first time the burden of OM in Aboriginal as well as non-Aboriginal children aged <2 years in such a setting and assess the use of TEOAEs in the first three months of life in predicting subsequent risk of OM before age 2 years.

## Methods

Details of the methods used in the study, socioeconomic and demographic characteristics and the completeness of follow-up are described elsewhere [22]. Briefly, between April 1999 and January 2003, children born in Kalgoorlie Regional Hospital to mothers intending to stay in the area for at least 2 years were recruited into the study. Following informed consent from mothers, 100 Aboriginal and 180 non-Aboriginal babies were enrolled. Multiple births, children with severe congenital abnormalities or those whose birthweight was <2000 g were not eligible. An initial evaluation was conducted in the home 1–3 weeks postpartum. Subsequently children were to be seen at ages 6–8 weeks, 4, 6, 12, 18 and 24 months.

### Assessment of ear health

Ear health was assessed by a variety of methods at different ages: measurement of TEOAEs in children aged <3 months, tympanometry from age 3 months onwards, clinical examination on at least 3 occasions before age 2 years, and assessment of hearing from age 6 months onwards.

#### Transient Evoked Otoacoustic Emissions (TEOAEs)

From April 2000 onwards, following training by the senior audiologist (SW), research assistants (RAs) measured TEOAEs in quiet surroundings during the scheduled visits at ages 1–3 and 6–8 weeks using an Echocheck TEOAE hand-held screener (Otodynamics, Hatfield, UK) [14,23]. Results of the test required no interpretation and were recorded as pass, fail or not valid, the last usually due to excessive environmental or subject noise. Valid TEOAE measurements on each ear were included when investigating the association between TEOAE and subsequent risk of OM. However, to determine the prevalence of failed TEOAEs, if the TEOAE was not valid in one ear the overall TEOAE assessment for a child was documented as not valid. Babies who had two consecutive failed TEOAE responses were referred to an audiologist.

#### Specialist clinical examination

ENT/audiology clinics were held 4 times annually in the Audiology Department of the Kalgoorlie Regional Hospital for routine examination of study participants. Children were to have a clinical examination at least once at ages <6, 6–11 and 12–23 months. During the first year of the study, children were to be assessed more frequently by the resident audiologist (KM) during field follow-up visits, but subsequently there was no audiologist permanently resident in Kalgoorlie. We asked parents and guardians whether the child had a cold or had any current ear problems. For convenience, children were occasionally seen by an ENT specialist (FL) at clinics held every 2–3 months at Bega Garnbirringu Aboriginal Health Services Aboriginal Corporation (BEGA).

The ENT specialists established a clinical diagnosis using otoscopy, pneumatic otoscopy and tympanometry. Diagnosis was based on national clinical guidelines [24] and classified as normal, eustachian tube dysfunction, OME, AOM without perforation, AOM with perforation, dry perforation, perforation with purulent discharge, or unknown when complete examination was not possible (e.g. unable to visualise TM). It was not possible to make a diagnosis of chronic suppurative OM given the extended time intervals between clinical examinations. The final overall clinical diagnosis was based on the child's most severely affected ear. If a diagnosis could not be made for one ear (e.g. due to presence of wax), then the final diagnosis was based on the diagnosis for the other ear. However, to examine the association between failed TEOAEs and subsequent risk of OM, we included all available diagnoses on each ear.

Children were followed up, treated or referred by the ENT specialist or audiologist as required. Routine and review visits were clearly differentiated on the database. Here we present only results of routine clinical examinations. But, when reporting on the number of children who had TM perforations, we have included additional information obtained from local medical practitioners, with parental consent obtained to access their medical records [22].

#### Tympanometry

Tympanometry with the 226 Hz probe tone is generally not recommended before age 6 months since a compliant ear canal may result in lower sensitivity of the test. However, good specificity and positive or negative predictive values have been reported in young children [25]. In view of irregular examinations of young children by a medical specialist, we did tympanometry from age 3 months during routine clinic and field visits to obtain an estimate of burden of OM in young infants, acknowledging that prevalence rates might, if anything, be underestimated.

Audiologists performed tympanometry (Grayson-Stadler GSI 38, Madison, Wisconsin, USA) on ears without discharge at the ENT clinic. Some children did not attend ENT clinics at all or only infrequently, despite assistance with transport and attempts to make appointments at the most convenient time for families. This might have resulted in a selection bias of those who chose to attend the routine ENT clinics. Therefore, to obtain additional, possibly less biased, ear health outcomes more frequently on more children, RAs performed tympanometry, using a Maico I24 screening tympanometer (Maico Diagnostics, Eden Prairie, MN, USA), during routine follow-up visits from May 2000 onwards, following training by an audiologist (SW). Inter-observer variation, involving two RAs doing tympanometry sequentially on the same child, was assessed regularly. All tympanograms were classified by an ENT specialist (HC) or an audiologist (SW) according to standard criteria [15,26]. If tympanometry could not be assessed in one ear, then the classification for tympanometry was based on the result for the other ear.

#### Hearing assessment

An audiologist performed hearing assessments in children aged 12–23 months at the routine ENT/audiology clinics. From March 2002 onwards, hearing was also assessed when children were aged 6–11 months. Conditioned Orientation Response Audiometry was conducted in a single wall paediatric test booth. Narrow-band filtered noises at 500, 1000, 2000 and 4000 Hz were presented by a GSI 16 Clinical Audiometer (Grayson-Stadler, Madison, Wisconsin, USA) calibrated to ANSI 1980 standards [15]. Responses were categorised by averaging the four frequencies tested and classified as normal (<= 25 dB HL), mild hearing loss (26–40 dB HL), moderate loss (41–60 dB HL), or severe loss (> 60 dB HL).

#### Analysis

The chi-square test with continuity correction and Fisher's Exact test were used to compare variables of interest in terms of TEOAE outcome. Logistic regression, incorporating Generalised Estimating Equations to account for repeated measures on individuals, was used to compare groups for tympanometry and hearing loss outcomes. The regression models were adjusted for age.

We used the Cox proportional hazards model to investigate progress to OM by computing hazard ratios for TEOAE failure. Survival times were from date of test to first subsequent OM diagnosis. The analysis was conducted on data from individual ears and robust standard errors were used to account for within-person correlation.

#### Ethical clearance

The study was endorsed by two local Aboriginal organisations in Kalgoorlie, namely BEGA and Ngunytju Tjitji Pirni Inc. Ethical approval for the study was given by the WA Aboriginal Health Information and Ethics Committee, the Ethics Committee of Princess Margaret Hospital in Perth, and that of the Kalgoorlie-Boulder Health and Education Region.

## Results

### Transient evoked otoacoustic emissions

**Figure 1:**
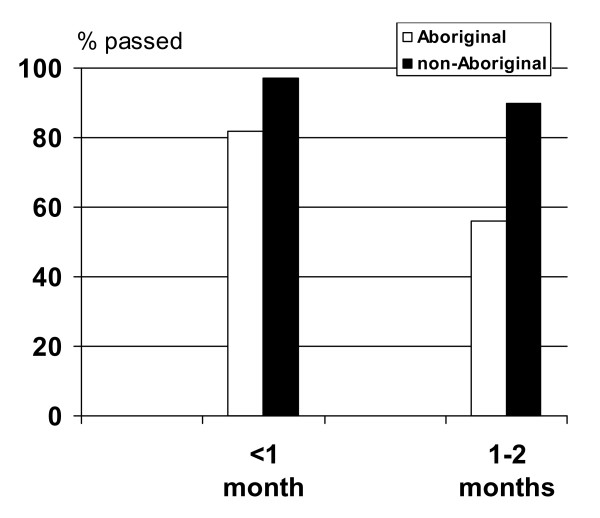
Proportion of Aboriginal and non-Aboriginal children with TEOAE responses present in both ears at ages <1 month and 1–2 months.

180 infants (57 Aboriginal and 123 non-Aboriginal) were screened at age <1 month and 168 infants (45 Aboriginal and 123 non-Aboriginal) were screened at 1–2 months. TEOAE responses were inconclusive for 6 (11%) Aboriginal children and 2 (2%) non-Aboriginal children aged <1 month and 11 (24%) Aboriginal and 7 (6%) non-Aboriginal children aged 1–2 months. Excluding children with non-valid results in one or both ears, TEOAE responses were present in at least one ear in 90% (46/51) of Aboriginal children and 99% (120/121) of non-Aboriginal children aged <1 month. Equivalent figures in the 1–2-month age group were 62% (21/34) and 93% (108/116), respectively. Figure 1 shows the proportion of children in whom TEOAE responses were detected in both ears. Pass rates were significantly lower in Aboriginal than non-Aboriginal children (<1 month 82% vs 97% Fishers Exact test p = 0.003; age 1–2 months 56% vs 90%, Yates chi-square = 17.33, p < 0.0001) (Figure 1). No children had sensorineural hearing loss.

### Tympanometry

Overall, 37% (n = 57) of 155 tympanometry readings that RAs conducted on Aboriginal children during routine follow-ups in the field were normal (type A), 50% (n = 78) had evidence of middle ear effusion (type B) and 13% (n = 20) had eustachian tube dysfunction (type C) compared with 62% (n = 296), 20% (n = 95) and 18% (n = 84), respectively, in 475 tympanometry readings in non-Aboriginal children (chi-square = 54.3, 2 df, p < 0.0001). There was a significantly higher prevalence of type B tympanograms in Aboriginal children than non-Aboriginal children both when done by RAs in the field (odds ratio (OR) = 4.35, 95%CI 2.73–6.95) and when done by audiologists at routine ENT clinic examinations (OR = 5.16, 95%CI 3.12–8.52) (Table 1). Type B tympanograms were recorded more frequently at routine ENT clinics than at routine follow-ups in the field in both Aboriginal (OR = 1.86, 95%CI 1.22–2.86) and non-Aboriginal (OR = 1.47, 95%CI 1.06–2.04) children. When we excluded the 21% of measurements in non-Aboriginal children whose parents reported current symptoms at the routine clinic visit, the prevalence of type B tympanograms was lower (15%, 26%, 22%, 24% and 25% at ages 3–4, 5–9, 10–14, 15–19 and 20–24 months, respectively) and closer to the field visit rates shown in Table 1. There was no such reduction in prevalence of type B tympanograms at routine clinics when we excluded the 14% of measurements in Aboriginal children who were symptomatic at the routine clinic.

**Table 1:** Classification of tympanograms in Aboriginal and non-Aboriginal children by age during routine follow-up by research assistants in the field or by audiologists at routine ENT follow-up.

Age (months)	Tympanogram	Aboriginal		Non-Aboriginal	
		Field	Clinic	Field	Clinic
			
3–4	A	13 (52%)	5 (33%)	65 (77%)	17(68%)
	B	11 (44%)	8 (53%)	9 (11%)	5(20%)
	C	1 (4%)	2 (13%)	11 (13%)	3(12%)
					
5–9	A	11(36%)	8(22%)	66 (61%)	47(56%)
	B	18 (58%)	26(72%)	30 (27%)	26(31%)
	C	2 (6%)	2(6%)	13 (12%)	11(13%)
					
10–14	A	15 (40%)	7(19%)	63 (54%)	31(49%)
	B	18 (49%)	25(69%)	26 (22%)	18(29%)
	C	4 (11%)	4(11%)	27 (23%)	14(22%)
					
15–19	A	11 (39%)	5(22%)	48 (60%)	36(52%)
	B	13 (46%)	15(65%)	16 (20%)	21(30%)
	C	4 (14%)	3(13%)	16 (20%)	12(17%)
					
20 +	A	7 (21%)	2(20%)	54 (63%)	8(44%)
	B	18 (53%)	6(60%)	14 (17%)	5(28%)
	C	9 (26%)	2(20%)	17 (20%)	5(28%)

The peak prevalence of type B tympanograms in both field and clinic was at age 5–9 months in both groups of children (Table 1). More than two-thirds of readings in Aboriginal children aged 5–19 months attending the routine follow-up clinic and approximately half in the field were type B. In non-Aboriginal children the prevalence of type B tympanograms was 25% between ages 5 and 14 months on examination in the field and 30% at routine ENT examination. Type C tympanograms were generally more common in non-Aboriginal than Aboriginal children (Table 1).

### ENT specialist examinations

Among the 83 Aboriginal clinic attenders 59% were male and 51% of the 164 non-Aboriginal attenders were male. Eighty-three percent of Aboriginal children were seen at least once for routine ENT follow-up and 59% were seen at least twice. Equivalent figures for non-Aboriginal children were 91% and 72%, respectively (Table 2).

**Table 2:** Number of times children attended ENT/audiology clinic for routine follow-up.

Frequency of routine follow-up at ENT clinic (%)								
	0	1	2	3	4	5	6	Total

Aboriginal	17 (17)	24 (24)	34 (34)	14 (14)	6 (6)	4 (4)	1 (1)	100
Non-Aboriginal	16 (9)	35 (19)	64 (36)	42 (23)	15 (8)	5 (3)	3 (2)	180
Total	33 (12)	59 (21)	98 (35)	56 (20)	21 (8)	9 (3)	4 (1)	280

Note: During the first year of study, children were seen by the visiting ENT specialist or by an audiologist who was resident in Kalgoorlie and examined children at time of field follow-up. Thereafter, children were to be seen routinely by an ENT 3 times before age 2 years.

**Figure 2:**
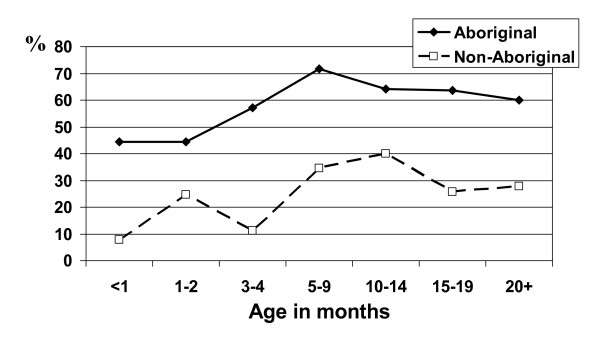
Age-specific prevalence of OM in Aboriginal and non-Aboriginal children on routine examination by ENT specialist.

On 184 routine clinical examinations in Aboriginal children between 8 days and 24 months of age, 55% had signs of OME, AOM or TM perforation (with or without purulent discharge); 27% of examinations were normal (Table 3). In non-Aboriginal children, 26% of 392 clinical examinations between age 6 days and 23 months had evidence of OME or AOM and 57% were normal (Table 3). In Aboriginal children, the prevalence of OM (i.e. OME, AOM, and/or perforations) rose from 44% in the first month of life to 72% at age 5–9 months and remained at 60% or more; in non-Aboriginal children, prevalence rose to 40% at age 10–14 months and was still 28% in those aged 20 months or more (Figure 2). Age-specific prevalence rates of OM were similar when examinations of symptomatic children were excluded.

**Table 3:** Clinical diagnosis by ENT specialists at routine follow-up.

Middle ear diagnosis	Aboriginal*		Non-Aboriginal*	
		
	N	%	N	%
Normal	49	26.6	225	57.4
Eustachian tube dysfunction	17	9.2	37	9.4
Otitis media with effusion	82	44.6	91	23.2
Acute otitis media (AOM)	4	2.2	9	2.3
AOM with TM perforation	9	4.9	2	.5
TM perforation ± discharge**	6	3.3	-	-
Other	2	1.1	3	.8
Unknown	15	8.2	25	6.4
Total	184	100.0	392	100.0

* 11% and 8% of diagnoses in Aboriginal and non-Aboriginal children, respectively, were based on successful examination of one ear only.

** 2 had dry TM perforation

A total of 21 (21%) Aboriginal children in the study had a TM perforation documented at least once during the study, the earliest being documented in an 8-day-old child. By the age of 6 months 7% of Aboriginal children had had a TM perforation at least once (6 of the 85 children followed up to age 6 months or more) and 19% (15/80) by the age of 12 months. A perforation was seen in 6 (3%) of the non-Aboriginal children.

ENT specialists recommended insertion of ventilation tubes (for recurrent AOM and/or persistent OME with hearing loss and speech concerns) in 12% (n = 12) of Aboriginal children and 10% (n = 18) of non-Aboriginal children. This provides a further indication of the burden of severe middle ear disease.

### Hearing assessment

**Figure 3:**
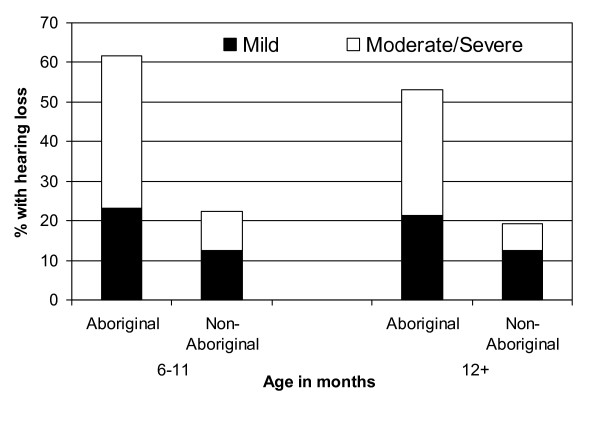
Prevalence of moderate-severe and mild hearing loss in Aboriginal and non-Aboriginal children aged 6–11 and 12–24 months of age.

Between 6 and 24 months of age, 61 routine hearing assessments were performed in Aboriginal children and 169 in non-Aboriginal children. Hearing loss was significantly more common in Aboriginal than non-Aboriginal children (OR = 5.40, 95% CI 2.68–10.89). In Aboriginal children moderate-severe hearing loss was seen in 39% of 13 assessments done at ages 6–11 months and in 32% of the 47 assessments in children aged 12 months or more (Figure 3). In non-Aboriginal children, moderate-severe hearing loss was detected in 10% of 40 assessments at age 6–11 months and in 7% of 120 assessments at age 12 months or more (Figure 3).

### TEOAE responses and subsequent risk of OM

We had valid TEOAE measurements in the first month of life followed by at least one successful clinical examination for 102 ears in 54 Aboriginal children and for 234 ears in 120 non-Aboriginal children; at age 1–2 months there were valid TEOAEs and subsequent clinical examination on 60 ears in 34 Aboriginal children and on 218 ears in 111 non-Aboriginal children. In Aboriginal children, OM was subsequently diagnosed in 55% (n = 46) of ears for which TEOAE responses were present <1 month of age compared with 72% (n = 13) of ears with no detectable TEOAEs at the same age. Equivalent figures for non-Aboriginal children age <1 month were 31% (n = 70) and 40% (n = 2), respectively. In 1–2-month-old Aboriginal children, 51% (n = 19) of ears with TEOAE responses present had a subsequent diagnosis of OM compared with 87% (n = 20) of those with no TEOAE detected. Equivalent figures for non-Aboriginal children were 37% (n = 73) and 32% (n = 6), respectively.

There was a non-significant increased risk of subsequent OM in all children with failed TEOAE before age 1 month (Table 4). In contrast, Aboriginal children who failed TEOAE at age 1–2 months were 2.6 times more likely to develop OM subsequently than those who passed TEOAE. Failed TEOAE response did not predict subsequent OM in non-Aboriginal children aged 1–2 months (Table 4). Results were very similar when examining TEOAEs in early infancy and subsequent observation of a type B tympanogram either on routine field follow-ups or at routine ENT clinics (data not shown).

**Table 4:** Hazard ratios (and 95% confidence intervals) of OM among Aboriginal and non-Aboriginal children with no detectable TEAOE response, compared with those with a TEOAE response, at ages <1 month and 1–<3 months

Age	Aboriginal		Non-Aboriginal	
		
	Hazard ratio	95% CI	Hazard ratio	95% CI
<1 month	1.19	(0.66 – 2.16)	2.51	(0.38 – 16.48)
1–<3 months	2.64*	(1.32 – 5.31)	0.71	(0.24 – 2.11)

* p = 0.006

## Discussion

To our knowledge this is the first comprehensive investigation of middle ear health (which includes hearing assessment) conducted simultaneously in young indigenous and non-indigenous children living in an urban setting, although an Australia-wide study of Aboriginal and non-Aboriginal people of all ages was conducted 30 years ago [5]. All study participants had high rates of OM but rates were particularly high in Aboriginal children, in whom disease began at a very young age, as has been reported previously [7,9,10]. There was significant hearing loss from age 6 months onwards, particularly in Aboriginal children, one-third of whom had hearing loss >40 dB. The general lack of symptoms with such high disease burden is of concern since families would not be prompted to bring children for medical care.

Aboriginal children were more likely to fail TEOAE measurements than non-Aboriginal children in the first 3 months of life, which is consistent with the earlier onset of OM in Aboriginal children. Of particular interest was the finding that absent TEOAEs in Aboriginal children at age 1–2 months predicted subsequent risk of OM. Not surprisingly, such an association was not seen in non-Aboriginal children, given their high pass rate and later onset of disease.

### Comparison with other studies

*Prevalence *The burden of OM and age-specific prevalence in non-Aboriginal children is comparable to that found in studies undertaken elsewhere [1,27,28]. In Aboriginal children, the age-specific prevalence of OM, and specifically the prevalence of TM perforations, in this urban/periurban semi-arid area of WA is lower than in many communities in the NT and WA, though prevalence of disease varies widely between communities [11,29,30]. The lower prevalence of OM in the Kalgoorlie-Boulder region of WA compared with that reported in many NT Aboriginal communities is consistent with lower bacterial carriage rates [9,31]. One cannot exclude the possibility that selection bias may have contributed to the lower rates of disease in our study, i.e. that parents of a healthier group of children chose to participate in our study.

While there are no directly comparable data in WA, the degree of hearing impairment in Aboriginal children in our study is consistent with that reported among children aged <5 years in three Aboriginal communities in 1988–89, where the prevalence of hearing loss ranged between 38% and 63% [29], suggesting that there has been little improvement in ear health in the past 20 years.

In a state-wide population-based study of Aboriginal children in WA 25% of children aged <3 years living in areas of moderate isolation (such as the Kalgoorlie-Boulder region) had a history of ear discharge [13], consistent with our finding that 21% of Aboriginal children had one or more perforations by age 2 years. It is interesting, however, to note that 12% of Aboriginal children in our study were referred for insertion of ventilation tubes. This suggests that, in urban areas at least, closed ear disease (as opposed to perforated ear drums) may now be more common than in the past.

Hunter *et al *conducted a cohort study among American Indians using a similar design to ours, with regular follow-up of 366 children from birth to age 2 years [21]. The prevalence of OM by examination of individual ears was lower (14%, 31%, 47% and 33% in children aged <2, 2–5, 6–12 and 13–24 months, respectively) than in our Aboriginal study population. In the same study, excluding examinations with technical failures, the failure rate of distortion product OAEs (DPOAEs) in American Indian children was of the same order as the TEOAE failure rate in Aboriginal children in our study: 25% at age <2 months and 41% at age 2–5 months compared with 18% < 1 month and 44% age 1–<3 months in Aboriginal children in our study.

### Otoacoustic emissions

The pass rate for TEOAEs of 97% among non-Aboriginal children in our study is consistent with the pass rate of a newborn hearing screening in Perth, WA, in which the pass rate was 99% following assessment soon after birth and a repeat test if the baby failed the first time [23]. To our knowledge there are no published studies that have measured OAEs with comparable study design in healthy young non-indigenous children after the early neonatal period.

Our TEOAE measurements in Aboriginal children were inconclusive in 11% of children aged <1 month. This is higher than the reported 5% technical failure rate in American Indian children aged <2 months [21], but the difference is not statistically significant. In older Aboriginal children the proportion of TEOAE tests that were inconclusive was 24%, similar to a technical fail rate of 27% in American Indian children aged 2–5 months [21].

*OAE as predictor *Doyle *et al *[20] followed a small number of children with and without middle ear effusion (MEE) at birth. Investigators performed otoscopy in addition to TEOAE measurement and found that early onset of MEE predicted subsequent OME and hearing loss in the first year of life [20]. The only known published study in an indigenous population reported no association between failed TEOAE measurements in newborn American Indians and risk of recurrent OM before age 2 years [21], though the outcome measures differed between the two studies ('recurrent OM' as opposed to time to first diagnosis of OM in our study). We also found no association between failed TEOAE in the first month of life and subsequent risk of OM, but such an association was present for TEOAE measurements after the neonatal period. To determine whether our findings can be generalised to other indigenous populations, the use of TEOAE measurements as a predictor should be evaluated in other settings.

### Strengths and limitations of the study

The strength of our study is that it provides new information on the burden of OM, including impaired hearing beyond the first year of life and detection of TEOAEs in indigenous and non-indigenous children living in the same region. Furthermore, it is the first study in Australia to investigate TEOAE as a predictor of subsequent disease. The activities around this study also raised awareness about OM and the ENT specialist provided expertise not only to study participants but to others as requested at a time when limited ENT services were available in Kalgoorlie [22].

The principal aim of our cohort study was to investigate microbiological, immunological, demographic and socioeconomic factors predisposing to OM. In view of the infrequent assessments of middle ear status, we were unable to follow the natural history of the disease and determine whether the hearing loss and presence of middle ear effusions were continuous or intermittent, as has been described elsewhere [32].

Data are limited for some outcomes. In particular a limited number of Aboriginal children were seen by the ENT specialist on all 3 intended visits. However, tympanometry during routine field follow-up provided important supplementary information on middle ear health and confirms the enormous burden of disease, particularly in Aboriginal children. Associations between failed TEOAEs and presence of type B tympanograms were consistent between field and clinic follow-up measurements.

### Recommendations for surveillance

Given the high prevalence of asymptomatic OM, particularly in Aboriginal children, regular surveillance for ear disease and hearing loss must be rigidly applied. This implies first and foremost strong financial support for primary health care, as well as training and supervision of primary health care staff.

An optimal screening program in infants should include newborn hearing screening, followed by otoacoustic emission testing or high-frequency tympanometry at age 1–2 months to identify children at increased risk of subsequent OM, and then audiometry and tympanometry (with 226 Hz probe tone) between the ages of 6 and 12 months. Services need to be available so that children can be referred according to national guidelines [24]. Aboriginal Health Workers (AHWs), nurses and doctors need to be encouraged to do otoscopy and/or tympanometry whenever a child presents with URTI and/or fever or irritability in order to initiate treatment at an early stage of disease [12,24].

Following on from our study, an ear health screening program is being introduced in the Goldfields region. The program includes training of community health nurses and AHWs in tympanometry, otoscopy and audiometry. Children are to be assessed at birth, 1–2 months, then 6-monthly from 6–18 months and annually thereafter to 5 years. Treatment and referral procedures are according to local standardised protocols based on national guidelines [24]. This program needs full support and should be formally evaluated. Such an evaluation should consider detection and referral rates, acceptability of the program by health service providers and families and changes in disease rates over a 5-year period as well as an economic evaluation of the program.

### Recommendations for research

1. The role of health professionals (e.g. nurses and AHWs) with specialist training in ear health should be formally evaluated as anecdotal evidence suggests that such models have been successful in identifying and treating children at Aboriginal Medical Services (Derbarl Yerrigan Aboriginal Medical Service in Perth) and in New Zealand (Variety Ear Bus Program).

2. Children who participated in our study are now aged 5 – 9 years and of school age. It would be worthwhile identifying those still in the Kalgoorlie-Boulder region to determine the long-term outcomes with regard to hearing, speech, language and education, based on our initial clinical assessments of middle ear health.

3. Measurement of TEOAEs at age 1–2 months to identify those at risk of developing OM should be evaluated in a routine health service setting (e.g. through an Aboriginal Medical Service and/or Ngunytju Tjitji Pirni Inc, a Kalgoorlie-based Aboriginal maternal and child health service provider). This could coincide with the two-month immunisation visit. Audiological services must, however, be available for referral of children who fail TEOAE on two occasions for appropriate management. Such a study is currently in the planning stage.

4. As part of the ear health program in the Goldfields, we propose a study comparing performance of TEOAE measurement with that of high-frequency tympanometry at age 1–2 months in predicting risk of subsequent OM. The study will include an economic component and we will ask primary health care workers to comment on the practicality of the different techniques.

5. Given the high rates of OM in the Aboriginal population, evaluation of an intervention addressing hygiene practices to reduce upper respiratory bacterial carriage and hence OM is urgently needed. This must not deter from the need to increase availability of appropriate housing to reduce transmission of respiratory pathogens in crowded homes.

6. The currently available 7-valent pneumococcal conjugate vaccine has not reduced the burden of OM in Aboriginal children in NT [33,34]. There is, however, another conjugate pneumococcal vaccine linked to *Haemophilus influenzae *protein D, which has been found to be efficacious in preventing episodes of acute OM in Czech Republic [35] and merits evaluation in the Aboriginal population. Maternal immunisation with 23-valent pneumococcal polysaccharide vaccine for prevention of OM in their offspring is currently being evaluated in the NT. Other protein-based vaccines to prevent OM due to the pneumococcus, *H. influenzae *and *Moraxella catarrhalis *are under investigation.

## Conclusion

In summary we have found high rates of OM in generally asymptomatic Aboriginal and non-Aboriginal children in an urban/periurban setting in a semi-arid zone of Australia, the rates being particularly high in Aboriginal children, though lower than reported in more remote settings. One-third of Aboriginal children over the age of 6 months had significant hearing loss. The absence of OAEs after the neonatal period in Aboriginal children confirms the early onset of middle ear disease and OAE measurement may be used to identify children at risk of developing OM. Given the silent nature of the disease, regular surveillance for OM and hearing loss must be applied frequently from a young age. The technology to measure TEOAEs using the Echocheck is less expensive than the alternative multi-frequency tympanometer in children under the age of 6 months. However, the recent availability of a screening tympanometer with a 1000 Hz probe tone option may now make tympanometry a more viable alternative option as, unlike OAE measurements, it does not require a quiet environment and settled child. The Echocheck is a simple screening tool and its use should be evaluated in a primary health care setting.

## Competing interests

The authors declare that they have no competing interests.

## Authors' contributions

DL established the study, designed questionnaires and supervised all aspects of the study and wrote the article for submission. PJ undertook data analysis and assisted in preparation of the manuscript. HC is an ENT specialist who conducted almost all the routine clinical examinations on study participants. SW conducted tympanometry and audiometry at routine ENT clinics, trained research assistants in tympanometry and wrote an early draft of the manuscript. HC and SW classified all tympanometry readings. DE oversaw all data management and field work. DE, AS, RM, JF provided input into development of questionnaires, conducted interviews with study participants, assisted in ensuring attendance at routine ENT clinics and undertook tympanometry during routine field follow-up. All authors have seen and approved the manuscript prior to submission.

## Pre-publication history

The pre-publication history for this paper can be accessed here:


